# Supplementary material for: Evolution of parasitism and mutualism between filamentous phage M13 and Escherichia coli
Source: PeerJ. 2016 May 24;4:e2060. doi: 10.7717/peerj.2060 (PMC4888304; doi:10.7717/peerj.2060)
Supplement: Supplemental Information 1 [file peerj-04-2060-s001.docx]

**Supplementary Material for “Evolution of parasitism and mutualism between filamentous phage M13 and *Escherichia coli*”**

Jason W. Shapiro, Elizabeth S.C.P. Williams, Paul E. Turner

*Discrete time model of phage persistence through vertical transmission*

In the study, we observed the loss of phage production in 15 of 18 populations undergoing vertical transmission, despite the observation that phage increased host growth rate and density. Past work observed similar phage loss under serial transfer when phage were moderately virulent (Merriam 1977). Phage production was also lost in work where viruses were engineered to provide antibiotic resistance (Bull & Molineux 1992). It has been suggested that phage loss is a normal part of filamentous phage life history and the result of low phage copy number within established infections and the absence of a mechanism for fair segregation of phage between bacterial daughter cells (Lerner and Model 1981). We propose that this same process may explain the loss of phage observed in the treatment with no host addition (Treatment N).

To test this hypothesis, we implemented the following discrete time SI model with logistic growth in R (equations provided here, with fully commented R code available through figshare DOI: 10.6084/m9.figshare.2066064)

$$S_{t+1}=S_{t}+R_{t}-\min\left( eS_{t}V_{t},R_{t} \right),$$

$where R_{t}=(rS_{t}+\left( 1-p \right)\left( r+b \right)I_{t})(1-\frac{S_{t}+I_{t}+N_{t}}{K})$

$$N_{t+1}=min(eS_{t}V_{t},R_{t})$$

$$I_{t+1}=I_{t}+(p\left( r+b \right)I_{t}+\left( r-c \right)N_{t})(1-\frac{S_{t}+I_{t}+N_{t}}{K})$$

$$V_{t+1}=max(V_{t}+\lambda\left( fI_{t}+N_{t} \right)-\min\left( eS_{t}V_{t},R_{t} \right),0)$$

*S* represents the number of susceptible hosts, *N* the number of newly infected hosts, *I* the number of established infections, *V* the number of viruses; *t* indicates a discrete time step; *r* is the intrinsic growth rate of all bacteria; *b* is the benefit of phage to growth in established infections; *c* the cost of phage in new infections; *e* is the intrinsic rate of infection (encompassing attachment rate and entry of phage into bacteria); *p* is the rate of phage retention in established infections; *λ* is the rate of phage production in new infections; *f* is the fraction of the phage production maintained in established infections, *K* is the carrying capacity.

Note: this model allows for the possibility that phage production declines in established infections, as has been observed (Merriam 1977), though this is not discussed in the main text. We also tested a parameter set in which new infections incur a slight reduction of growth rate, *c*, but this parameter had no significant impact on the phase diagrams and was later set so that new infections have the same intrinsic growth rate as established infections; only their rate of phage retention and production may differ. Further, we use the “min” and “max” functions to ensure that values are not driven to impossible values (e.g. negative numbers). This model also ignores any possible effects of phage on bacterial yield. We considered adding in net effects of phage on intrinsic death rate or changing competition coefficients within the logistic expression in the equations, but these changes are not necessary to get at the basic question of whether or not phage can go extinct when they increase bacterial growth rate.

To implement serial transfers, we added a conditional statement in the R code that first sums all bacteria at time *t*, and if that total exceeds 99% of the carrying capacity, *K*, all densities (*S, I, N, V*) are diluted by a dilution factor “dil” of 1:100 (0.01), as in the original experiments. (Note: in the original experiments dilutions occurred at 24 hour intervals, not based on reaching a defined density.)

We then ran this model for 1000 time steps and recorded the endpoint value for viral density (V_1000_). If this value was less than 0.5, we rounded down to 0, and if above 0.5, we set the value to 1. We then created the phase diagrams below to show how varying the benefit level, *b*, and the retention rate, *p*, affect the rate of phage extinction. Parameter values are summarized in Table S1.

In all iterations of the model, we use the intrinsic rate of infection *e =* 3 x 10^-11^, reported previously (Lerner and Model 1981). Figure S1 shows that phage extinction is possible for beneficial phage, even with retention rates above 90%. There was almost no noticeable effect of reducing the rate of production in established infections (as seen in Merriam 1977) or of allowing new infections to be less beneficial (or even harmful) to the hosts.

While this simple model does not account for evolution or many of the details of phage life history, it strongly indicates that high rates of retention are required to prevent the extinction of viruses that increase growth rate. As such, we should not be surprised that phage went extinct in Treatment N.

**Table S1. Parameter values used in the model**

| **parameter** | **description** | **value** |
| --- | --- | --- |
| *r* | intrinsic growth rate | 1 |
| *c* | net cost of new infection | -*b* or 0.5 |
| *b* | net benefit of old infection | from -0.25 to 0.25 (0.01 steps) |
| *K* | carrying capacity | 10^9^ |
| *e* | intrinsic infection rate | 3 x 10^-11^ |
| *λ* | phage production per host | 1 |
| *S_1_* | initial susceptible density | 10^6^ |
| *I_1_* | initial infected density | 0 |
| *N_1_* | initial new infected density | 0 |
| *V_1_* | initial phage density | 100 |
| *dil* | dilution factor | 1:100 (0.01) |
| *f* | reduction of phage production in old infections | 1 or 0.1 |

**
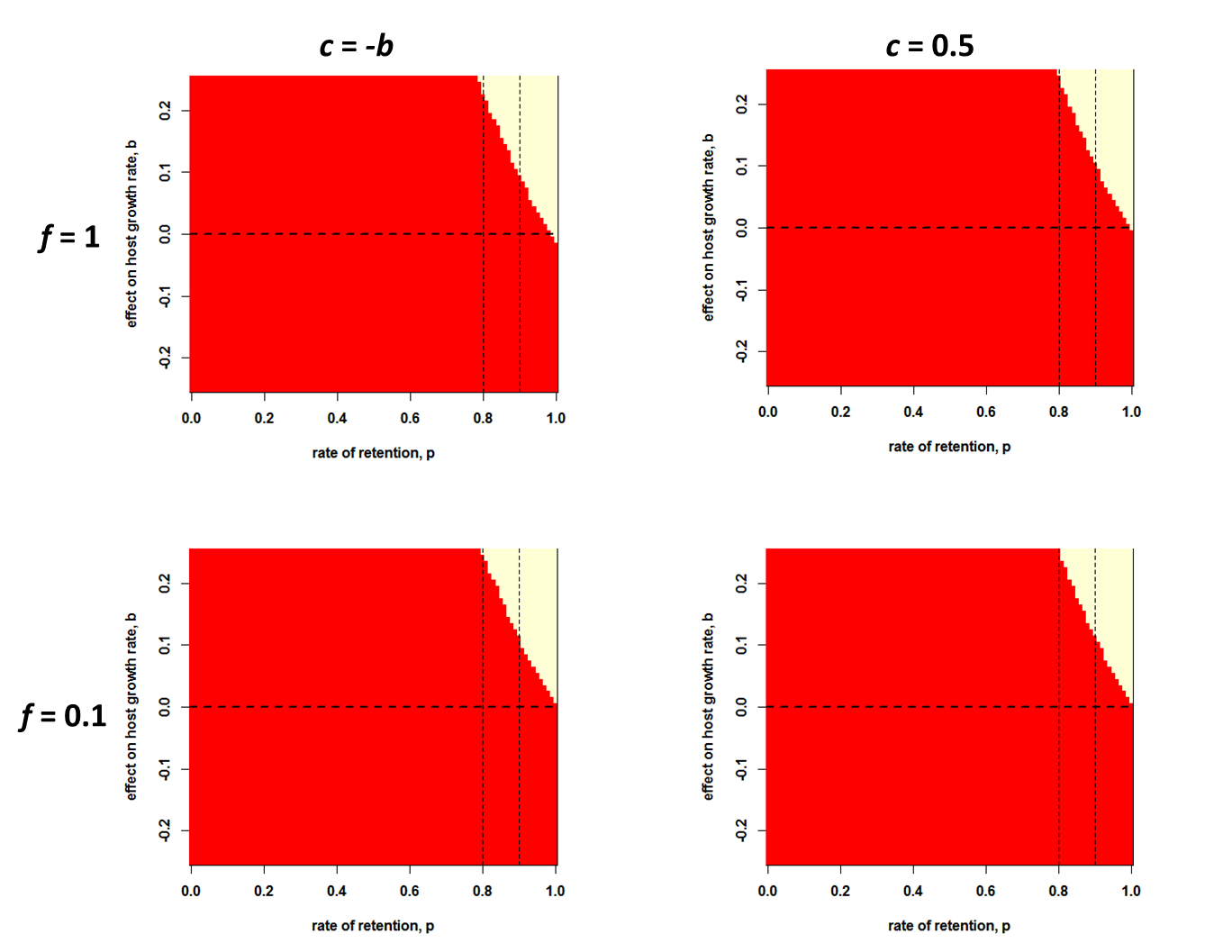
**

**Figure S1. Phase diagrams of phage extinction under varying benefits and retention rates**

Each panel shows the parameter space in which phage go extinct by generation 1000 (red area) or remain present (white area) under variation in the net effect on host fitness, *b* (y axis) and in the retention rate, *p* (x axis). The results are nearly identical when accounting for either reduced productivity in established infections (bottom row) or for greater costs of new infections to hosts (right column).

**References**

Bull, J. J., and I. J. Molineux. Molecular-Genetics of Adaptation in an Experimental-Model of Cooperation. Evolution. 1992; 46:882-895.

Lerner, T.J. and P. Model. The “steady state” of coliphage f1: DNA synthesis late in infection.

Virology. 1981; 115: 282-294.

Merriam, V. Stability of the carrier state in bacteriophage M13-infected cells. Journal of

Virology. 1977; 21 (3): 880-888.
